# Comparable and Complimentary Modalities for Treatment of Small-Sized HCC: Surgical Resection, Radiofrequency Ablation, and Microwave Ablation

**DOI:** 10.3390/jcm12155006

**Published:** 2023-07-29

**Authors:** Jeffrey S. Wicks, Benjamin S. Dale, Luis Ruffolo, Ludia J. Pack, Richard Dunne, Marie A. Laryea, Roberto Hernandez-Alejandro, Ashwani Kumar Sharma

**Affiliations:** 1 Department of Biology, University of Rochester, Rochester, NY 14642, USA; jwicks2@u.rochester.edu; 2 Department of Surgery, University of Rochester, Rochester, NY 14642, USA; benjamin_dale@urmc.rochester.edu (B.S.D.); luis_ruffolo@urmc.rochester.edu (L.R.); 3 Department of Genetics, University of Rochester, Rochester, NY 14642, USA; ludia_pack@urmc.rochester.edu; 4 Division of Hematology/Oncology, Department of Medicine, University of Rochester, Rochester, NY 14642, USA; richard_dunne@urmc.rochester.edu; 5 Division of Gastroenterology/Hepatology, Department of Medicine, University of Rochester, Rochester, NY 14642, USA; marie_laryea@urmc.rochester.edu; 6 Division of Transplant, Department of Surgery, University of Rochester, Rochester, NY 14642, USA; roberto_hernandez@urmc.rochester.edu; 7 Division of Interventional Radiology, Department of Imaging Sciences, University of Rochester, Rochester, NY 14642, USA

**Keywords:** HCC, RFA, microwave, surgical resection

## Abstract

Background: Over the past decade, there has been continual improvement in both ablative and surgical technologies for the treatment of hepatocellular carcinoma (HCC). The efficacy of ablative therapy compared to surgical resection for HCC has not been thoroughly evaluated using multiple large-scale randomized controlled trials. By international consensus, if a patient is eligible, surgery is the primary curative treatment option, as it is believed to confer superior oncologic control. Objective: to determine the efficacies of percutaneous ablative therapies and surgical resection (SR) in the treatment of HCC. Data sources, study appraisal, and synthesis methods: A meta-analysis using 5 online databases dating back to 1989 with more than 31,000 patients analyzing patient and tumor characteristics, median follow-up, overall survival, and complication rate was performed. Results: Ablative therapies are suitable alternatives to surgical resection in terms of survival and complication rates for comparable patient populations. For the entire length of the study from 1989–2019, radiofrequency ablation (RFA) produced the highest 5-year survival rates (59.6%), followed by microwave ablation (MWA) (50.7%) and surgical resection (SR) (49.9%). In the most recent era from 2006 to 2019, surgical resection has produced the highest 5-year survival rate of 72.8%, followed by RFA at 61.7% and MWA at 50.6%. Conclusions and key findings: Depending on the disease state and comorbidities of the patient, one modality may offer superior overall survival rates over the other available techniques. Interventional ablative methods and surgical resection should be used in conjunction for the successful treatment of small-sized HCC.

## 1. Introduction

Minimally invasive percutaneous interventions play an integral part in the treatment of hepatocellular carcinoma (HCC) in the modern medical landscape. By international consensus, the most effective treatment for HCC in a cirrhotic population is liver transplantation. However, in light of the less invasive procedures offered by interventional radiologists, paired with advances in technologies and techniques, surgery may no longer be the standard bridging therapy to transplant for small-sized (<5 cm) HCC [1,2]. This study was designed to compare percutaneous ablation to surgical resection. This did not include minimally invasive surgical techniques, such as laparoscopic surgical ablation. In the past, radiofrequency ablation (RFA) was the minimally invasive percutaneous standard offered to patients who were not surgical candidates [3].

The use of ablative therapies for the management of liver tumors began in the 1980s when microwave and radiofrequency ablation were first implemented in medical care [3]. Prior to the present study, it was unclear how their efficacy compared among radiofrequency ablation, microwave ablation, and the other currently existing mainstays of cancer management for small-sized HCC prior to transplant. Although there are now large data sets on patients treated around the world with MWA, RFA, and surgical resection (SR), there are few large-scale randomized studies or large-scale meta-analyses looking at the relative effectiveness of these modalities [4,5]. It should be noted there are many older and smaller analyses comparing the relative effectiveness of these modalities, many of which were used in our analysis if they fit the inclusion and exclusion criteria.

Naturally, prospective multicenter randomized trials will be required for large-scale validation. Multiple narrative and systematic reviews have been published that share data and are limited in a number of ways. Previous analyses are characterized by a lack of overall survival data, limited regional representation, small patient populations, few complication rates, and missing toxicity data [4,5,6,7,8,9,10,11]. In place of survival data, other metrics such as local recurrence, long-term progression, and disease-free survival were utilized. A meta-analysis is presented to help determine the optimal treatment of small-sized (<5 cm) HCC tumors by adopting overall survival and complication rates as outcome variables. The following article is in accordance with the PRISMA reporting checklist.

## 2. Materials and Methods

**Figure 1:**
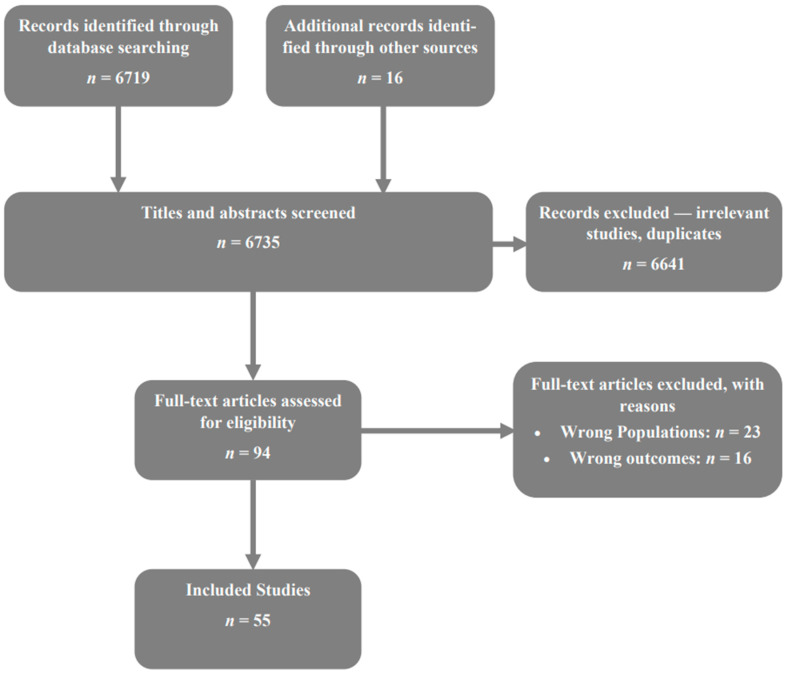
Flowchart of data collection process. A total of 6735 titles abstracts screened and 55 studies included in meta-analysis.

The Preferred Reporting Items for Systemic Review and Meta-Analyses (PRISMA) guidelines were followed for this multicenter meta-analysis. Five electronic databases (PubMed, EMBASE, Web of Science, EBSCO, and the Cochrane Library) were used to search for publications dating back to 1989 on ablative therapy and surgical resection. Keywords used were liver, cancer, HCC, microwave, radiofrequency, ablation, surgical resection, liver resection, and hepatocellular. All search results were reviewed, and their reference lists were analyzed to identify additional studies. After pruning irrelevant studies and duplicates, two reviewers confirmed the inclusion and exclusion criteria were met [JSW, LJP]. No discrepancies with the original screening were found. Two investigators obtained and confirmed the presence of the same data sets. The investigators worked independently and upon dispute, reviewed the source data together [JSW, LJP]. No automation tools were used. When data were incomplete and unavailable to be extracted, the two investigators confirmed the absence of data in the source study. If the study met inclusion and exclusion criteria, the available data were extracted as reported in PRISMA 2009 flow diagram (Figure 1).

To be eligible for meta-analysis, a study had to contain the following criteria: (1) studies analyzing MWA, RFA, or SR treatment of HCC; (2) studies reporting minimum of 3-year overall survival data; and (3) studies reporting tumor sizes that do not exceed 5 cm.

A study was excluded if it contained: (1) case reports, letters, reports; (2) reviews; (3) patient number < 65; (4) multifocal disease; (5) publication prior to 1989; or (6) publication after 2020. Studies with fewer than 65 patients contained large outliers in patient outcomes. Studies prior to 1989 represent a different era of surgical and ablative practice and were not representative of patient treatment today.

The extracted data included: (1) the first author, the study design, the location, the sample size, and the year of publication; (2) the oncological characteristics of patients (size, location, metastatic character, portal vein thrombosis, number of HCC); (3) the trial outcomes—survival, tumor resurgence, and complication rates; and (4) age data of the patient population, Child–Pugh score, median follow up for ablation procedures.

Through the inclusion criteria, we analyzed studies with a minimum follow-up of 1 year to assess complications and survival in order to properly evaluate the disease states and the treatment modalities. This was defined by the length of time that passes prior to a bridge-to-transplant treatment. If no bridge treatment was offered, patients would be followed indefinitely due to the data from ablation and SR being unaffected.

### Statistical Analysis

The plausibility and completeness of extracted data were evaluated prior to combining them into their respective modalities. Weighted overall survival graphs and tables were used to analyze long-term survival for the entire meta-analysis time period. Additionally, survival rates were further analyzed in two time periods (1989–2005 and 2006–2019) to evaluate how treatments varied over time. Weighting by study was performed in order to directly compare patient outcomes from each treatment center and to remove potential bias. Results were reported with 95% confidence intervals. Statistical significance of similarity was measured with the Mann–Whitney U test and with pooled odds ratios. Heterogeneity of data was analyzed with I^2^ and Q tests with a *p* < 0.05 representing significance.

## 3. Results

In total, 14, 22, and 28 studies were identified that the met inclusion criteria investigating MWA, RFA, and SR, respectively. A total of 3725 patients were involved in the MWA meta-analysis, and 10,838 patients were involved in the RFA meta-analysis. A total of 17,770 patients were involved in the SR meta-analysis. Due to the large patient number from medical centers around the world, there are significant differences between patient populations, and thus heterogeneity exists in certain cases. The most frequent surgical complications were intraperitoneal bleeding, portal vein thromboses, intrahepatic hematomas, intraperitoneal infections, bile leaks, bilomas, bile duct injuries, liver abscesses, abdominal bleeding, intestinal perforations, and diaphragmatic hernias. The most frequent percutaneous ablation complications were intra-peritoneal bleeding, intractable pleural effusion, hemothoraces, portal vein thromboses, intra-hepatic hematomas, bile leaks, bilomas, liver dysfunction, bile duct injuries, liver abscesses, intestinal perforations, diaphragmatic hernias, and tumor implantations.

**Figure 2:**
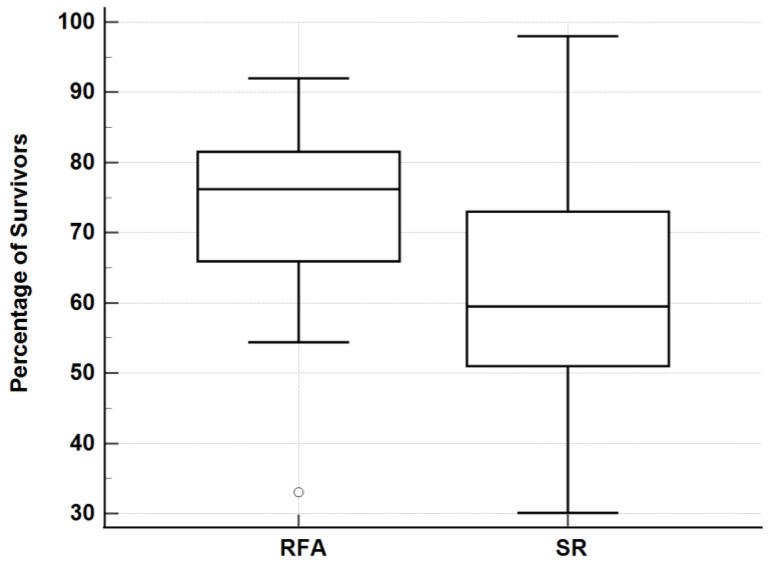
Three-year survival rates: RFA vs. SR. Mann–Whitney U test of 3-year survival rates for RFA and SR. Data presented as box plots. A higher median percentage and lower interquartile range observed by RFA. *p* = 0.0431.

**Figure 3:**
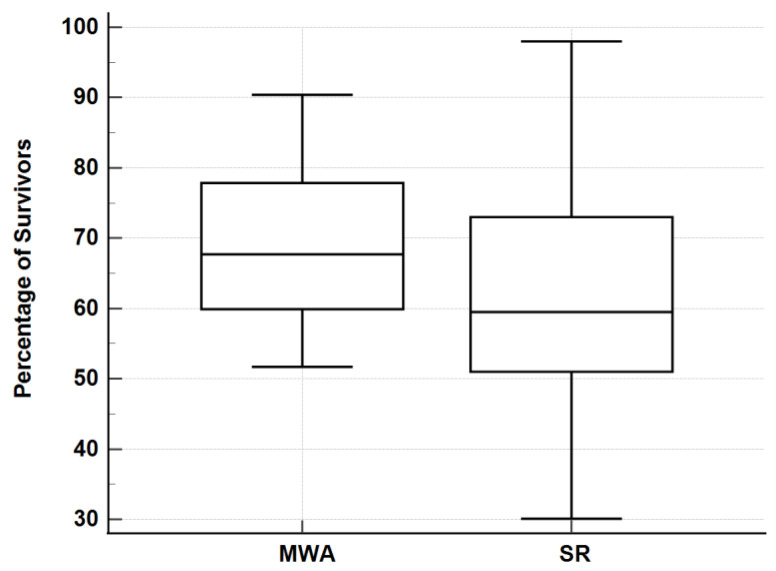
Three-year survival rates: MWA vs. SR. Mann–Whitney U test of 3-year survival rates for MWA and SR. Data presented as box plots. A higher median percentage and lower interquartile range observed by MWA. *p* = 0.2214.

**Figure 4:**
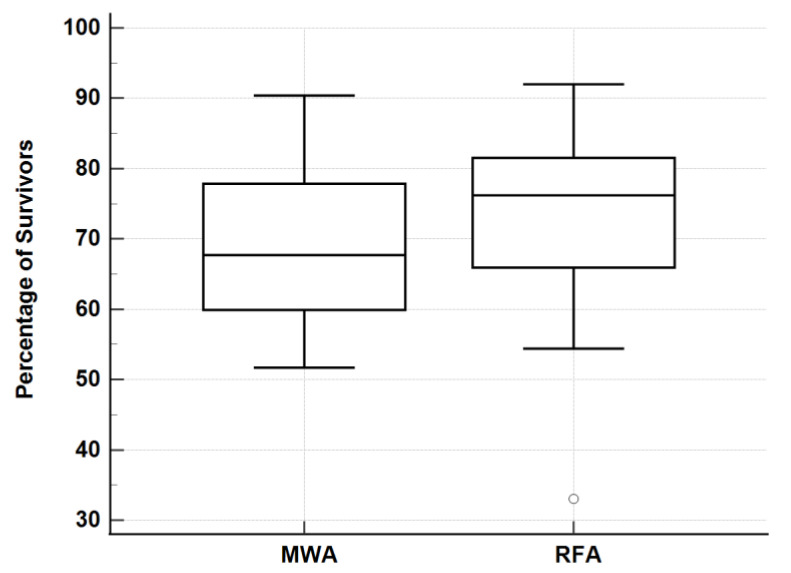
Three-year survival rates: MWA vs. RFA. Mann–Whitney U test of 3-year survival rates for MWA and RFA. Data presented as box plots. A higher median percentage was observed by RFA with a similar interquartile range observed by both modalities. *p* = 0.3930.

**Figure 5:**
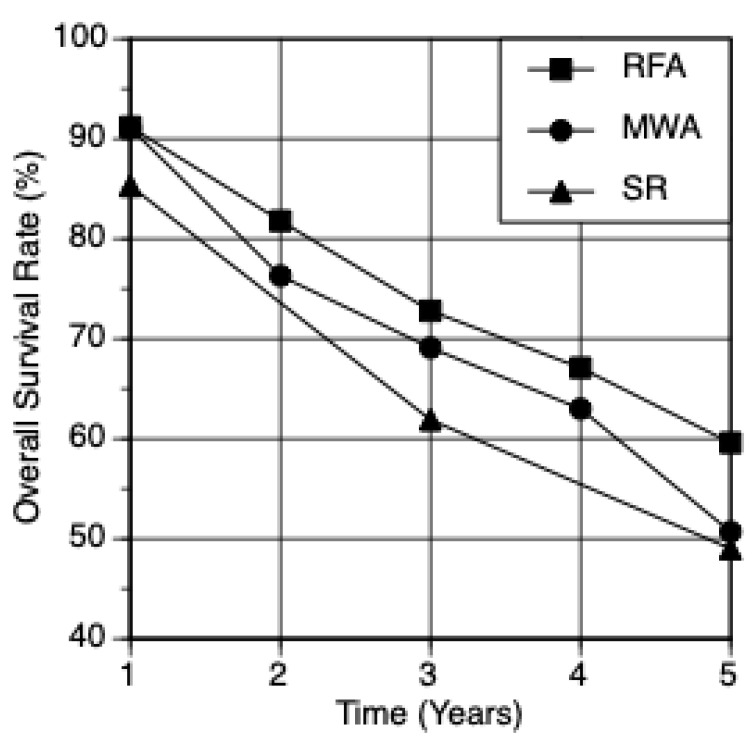
Comparison of Survival Rates: RFA, MWA, SR. The 1–5-year postoperative overall survival rate comparison; data weighted by study. RFA produced the highest survival rates, followed by MWA, while SR produced the lowest survival rates.

The comparison of meta-analyses showed that there was no significant difference in the survival and complication rates between the ablative techniques: MWA 1-,2-,3-,4-,5-year—91.1%, 76.3%, 69.1%, 63.0%, 50.7% with a 2.5% complication rate (Table 1); RFA 1-,2-,3-,4-,5-year survival—91.2%, 81.8%, 72.8%, 67.1%, 59.6% with a 3.4% complication rate (Table 2); SR 1-,3-,5-year—85.3%, 61.9%, 49.0% with a 32.9% complication rate (Table 3). When comparing survival rates, RFA and SR were significantly different (*p* = 0.0431) (Figure 2), MWA and SR were not significantly different (*p* = 0.2214) (Figure 3), and MWA and RFA were not significantly different (*p* = 0.3930) (Figure 4). The pooled odds ratio test confirmed this with *p* = 0.0001. The heterogeneity of data were confirmed with I^2^ and Q tests with Q = 1884 and *p* < 0.0001. The I^2^ value was 97.49%. The comparison of the ablative data to surgical resection showed an overlap in performance between modalities (Figure 2, Figure 3, Figure 4 and Figure 5) (Table 4).

In analyzing the full data set of the available studies (n = 55) and adjusting for study design, the treatment modalities were ranked based on overall survival rates as follows for the 1989–2019 time period: (1) RFA, (2) MWA, and (3) SR. For the same time period, the treatment modalities were ranked as follows based on complication rates: (1) RFA, (2) MWA, and (3) SR. Further subgroup analysis was conducted to evaluate the performance of SR and the ablative therapies by time period. In the 1989–2005 time period, the treatment modalities were ranked based on overall survival as follows: (1) MWA, (2) RFA, and (3) SR. In the 2006–2019 time period, the treatment modalities were ranked based on overall survival as follows: (1) SR, (2) RFA, and (3) MWA (Table 4).

When examining the effect of tumor size on survival rates, there were slight variations between each modality and tumor size; however, it can be clearly seen that tumors < 3 cm in size produced higher 5-year survival rates than tumors < 5 cm (Table 5). This is even more apparent with tumors < 2 cm in size. This can be seen across all modalities. SR produced the highest 5-year survival rates with tumors < 3 cm in size, whereas ablative therapies produced the highest survival rates on tumors < 5 cm in size, with RFA yielding the best survival rates in the category. For subgroup analysis, the treatments were broken into two categories—tumors < 5 cm but > 3 cm and tumors < 3 cm. For tumors < 5 cm but >3 cm treated with SR, the 1-, 3-, and 5-year survival rates are 80.3, 54.0, and 39.0 with a 34.4% complication rate. For tumors < 3 cm treated with SR, the 1-, 3-, and 5-year survival rates are 95.3, 88.4, and 69.4 with a 38.2% complication rate. For tumors < 5 cm but >3 cm treated with RFA, the 1-, 3-, and 5-year survival rates are 91.4, 68.6, and 55.6 with a 2.5% complication rate. For tumors < 3 cm treated with RFA, the 1-, 3-, and 5-year survival rates are 90.9, 76.6, and 62.21 with a 6.6% complication rate. For tumors < 5 cm but >3 cm treated with MWA, the 1-, 3-, and 5-year survival rates are 90.4, 61.4, and 43.5 with a 3.1% complication rate. For tumors < 3 cm treated with MWA, the 1-, 3-, and 5-year survival rates are 92.9, 82.0, and 57.8 with a 2.0% complication rate. The complication rates between tumor sizes are often comparable within each modality. It should be noted that ablative therapies vastly outperform SR for larger tumor sizes. In smaller tumor sizes, SR outperforms the ablative therapies at the cost of higher complication rates. However, all previous relative comparisons based on tumor size should be carefully analyzed with respect to the above statistical analysis measuring significance between modalities.

**Figure 6:**
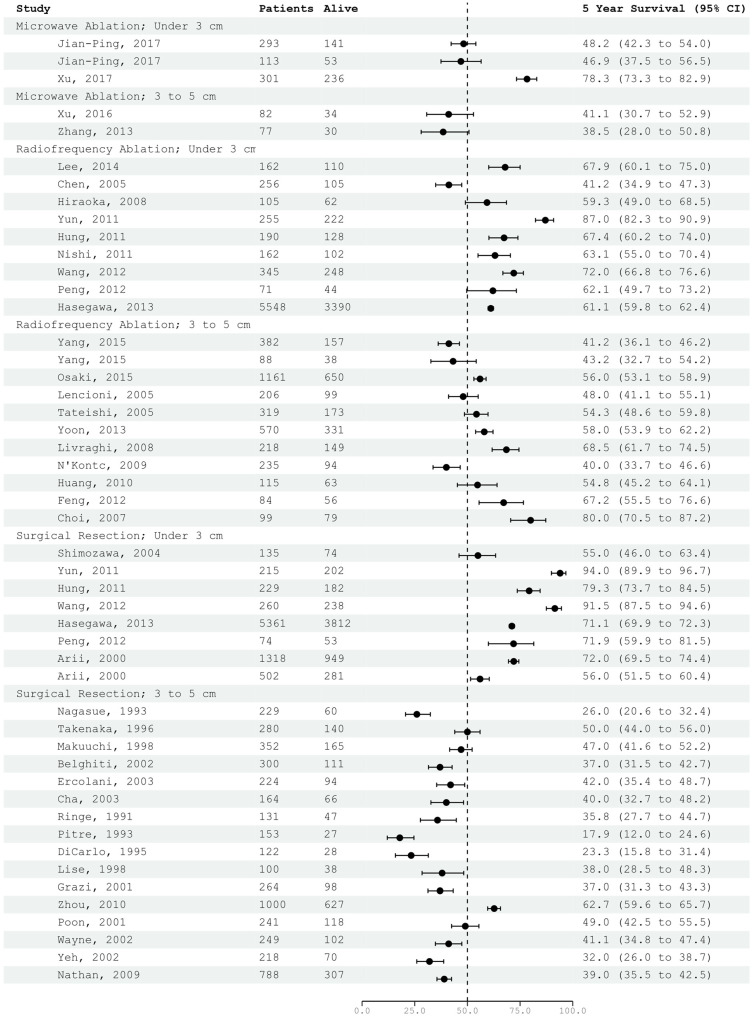
Forest Plot [1,6,7,14,15,17,18,19,20,21,22,23,24,25,28,29,31,32,33,34,35,36,37,38,39,40,41,42,44,45,46,47,48,49,50,51,52,54,55,57,58].

Figure 6 presents a comprehensive forest plot showcasing the 5-year survival outcomes and their corresponding 95% confidence intervals derived using a binomial confidence interval. The studies are categorized based on the therapeutic approach employed, specifically: microwave ablation, radiofrequency ablation, and surgical resection. Within each group, we performed further stratification based on the mean tumor size with the divisions being under 3 cm versus between 3 and 5 cm. Our analysis revealed a significant degree of heterogeneity among the studies, which is further validated by our forest plot. Remarkably, radiofrequency ablation demonstrates superior 5-year survival rates when compared to surgical resection. Additionally, across all therapeutic modalities, there appears to be a heightened efficacy observed in the treatment of tumors under 3 cm in size.

## 4. Discussion

This present meta-analysis details the advantages and disadvantages of varying modalities of treatment of HCC highlighted from the current literature, including RFA, MWA, and SR. It is apparent that many studies lack proper methodology, as the patient populations can be heterogenous in tumor features. This lack of uniformity is ubiquitous throughout both single-center studies as well as meta-analyses. It is exacerbated by different protocols from different regions around the world.

There are differences in treatment efficacy due to the limitations of surgical resection and ablative therapies. For example, due to tumor location or coagulation profile, there may be a risk of bleeding and peritoneal seeding. Similarly, tumor location may preclude complete necrosis. Conversely, patients with tumor locations that require high volumes of parenchymal sacrifice or patients with portal hypertension, compromised prothrombin time, low platelet count, and other liver dysfunction are excluded from surgery. Most medical centers have multi-disciplinary teams, which decide how to proceed in patient care. This enables a consensus among multiple professionals on how to treat a given disease state. Independent of varied results, there are reported instances in which ablation and surgery would both be viable options, allowing for a comparison. Additionally, these modalities are complementary to one another in that they excel in different circumstances. In the case of tumors that are <5 cm but >3 cm, ablative therapies offer superior efficacy. However, in the case of smaller tumors that are <3 cm in size, surgical resection provides greater efficacy.

From the meta-analysis, the overall survival rates show all studied modalities to perform well based on the selection of modalities driven by tumor characteristics. MWA is a relatively new modality, and as such, the data are not as extensive as RFA or SR. Even with fewer published data available, MWA produced similar overall survival rates as RFA. As more MWA studies are conducted and published, more data will be available to include in large-scale meta-analyses.

From the database search results, few meta-analyses and even fewer large-scale randomized control trials have been conducted to review the quality of surgical resection compared to ablative therapies [34,59,60]. The primary strength of the present meta-analysis is in the comparison of similar patient populations from around the world including more than 31,000 patients.

Based on BCLC criteria, poor candidates for surgery undergo ablative therapies [61]. In this study, candidate quality was quantified by (1) cancer stage; (2) size, number, presence of metastasis, location, and previous procedures performed; and (3) co-morbidities [62]. This study’s inclusion and exclusion criteria largely limited confusion associated with the combination of large data sets by comparing similar populations between modalities.

The three modalities produced complementary relationships with each other via the Barcelona clinic liver cancer (BCLC) staging, which uses a defined set of criteria to guide the care of patients with HCC. Depending on the stage of the liver disease, the location of the lesion, and the liver function, a decision can be made by the multi-disciplinary tumor board. Due to the nature of our meta-analysis including tumors <5 cm, the BCLC standards define the modality, which should be used. In this manner, the modalities complement each other for the optimal treatment of the patient.

This meta-analysis provides evidence in over 31,000 patients that surgical resection produced similar OS rates with higher complication rates compared to the ablative therapies of MWA and RFA. This is due to the specific character of each modality. RFA and MWA are minimally invasive percutaneous procedures that preserve the liver parenchyma as much as possible. As a result, the required hospital observation is short and can often be managed in an outpatient site [63]. The small length of required hospital observation is a strength of ablative therapies [11]. In this modern era of medicine, the ease of outpatient procedures is preferred by both patients and care providers alike [64,65]. SR is rapidly evolving in the creation of new protocols, including outpatient hepatectomies and minimally invasive hepatectomies (MIS), which will continue to decrease the length of stay post-operation. Additionally, these new protocols are more parenchymal sparing than previous techniques and protocols. It should be noted that these new surgical protocols and techniques yield significantly lower complication rates compared to the complication rates reported for the entire length of this study. In some cases, these new procedures have complication rates that are at parity with percutaneous ablative therapies.

Over the last 30 years, surgical resection has vastly improved in its efficacy in the treatment of HCC. Advances in medical technology paired with new techniques have enabled surgical resection to improve from the weakest performing modality among the three modalities to the strongest, with a 5-year survival rate increasing by 30% (from a 42% to 72% 5-year survival rate) in that time. RFA also saw an improvement in outcomes; however, it was not as drastic as the improvement SR saw. Throughout this analysis time period, MWA produced the same survival rates. These results for the entire time period of the meta-analysis can be counter to some smaller-scale recent studies of percutaneous ablation and surgical resection. To compare recent studies to the data in this meta-analysis, it is useful to focus on the 2006–2019 data where SR outperformed the other modalities in a 5-year survival rate.

For tumors <3 cm in size, if a patient is a surgical candidate, surgical resection yields the highest survival rates. For tumors < 5 cm in size, percutaneous ablative therapies provide superior survival rates. Due to the nature of the manner of reporting tumor sizes in the studies, we recognize a limitation in stratifying tumors as <5 cm and <3 cm. Naturally, tumors that are <3 cm in size are also <5 cm in size. However, we feel the data are useful, as <5 cm tumors include tumors that are larger than 3 cm, whereas tumors in the <3 cm category only contain tumors less than 3 cm in size. With that being said, a majority of the tumors in the <5 cm category are <3 cm in size. These stratifications and efficacies of treatment depending on modality match what the BCLC criteria dictate for patient care.

Although this study was a global analysis, useful conclusions were made from studying the different modalities in their prospective regions. For example, ablation data were limited in the United States, whereas in Korea, Japan, and China, there was an abundance of data, as these Asian nations have adopted ablative technology earlier than the West. Furthermore, in the 1980s when ablative medical technology was developed, Asia was the major production site for microwave and radiofrequency systems, and the infrastructure to develop MW and RF systems for medical ablation was in place [66]. While the Eastern world pushed ahead with ablation, the Western world mostly pursued traditional surgical resection. The result of this divergence was treatment quality varied by region. Compared with the global average, the East produced slightly superior ablation results, and the West produced slightly superior surgical results [33,45]. Furthermore, less healthy individuals (whether it be age, fitness, disease state, or comorbidities) are often not able to be candidates for surgery and thus undergo ablation, which is determined by the BCLC criteria and tumor boards. This should be considered when analyzing survival rates and complications.

Our study is a longitudinal analysis over the course of 20 years, and in some cases, the quality of a given modality has changed. This has resulted in some cases where recurrence for one modality may be worse in some studies but better in other studies. This is the result of such a large-scale analysis over this time period [67,68]. Shin et al. [67] showed liver resection is superior to RFA in terms of oncologic outcomes than MWA and RFA; they included studies more recent than our studies. However, we found the same result with tumors less than 3 cm (Table 5). Even though Takayama [68] et al. found a difference between RFA and surgery for the treatment of small HCC, we found clear differences among the different modalities depending on the size of HCC (Table 5). Wang [69] supported our observation of the clear advantage of ablation over surgery for tumors between 3–5 cm.

Determining the optimal treatment by comparing outcomes from medical centers across the world in their ability to perform MWA, RFA, and SR was the goal of this meta-analysis. There are previously existing smaller-scale analyses and studies on the treatment of HCC using SR, MWA, and RFA. These studies range in size from single medical centers to small multicenter hospital systems to smaller-sized countries and, in some cases, larger countries [5,12,23,35,38,53]. The value of our present analysis is the scale of multi-national global data collection, which yields insights not available from smaller studies. The comparison of the previously conducted smaller-scale studies with our large-scale study shows agreement in the outcomes, and thus a fuller consensus can be made on how best to treat HCC.

The overall-survival and complication rate data were weighted by study in order to directly compare the quality of care provided at each institution. This comparison was used as an instrument to account for differences among medical centers. As a result, these pooled data sets achieved an extraordinarily large sample size. This provided a view of MWA vs. RFA vs. SR with as large of a pool of data as possible. The data can be weighted or subdivided in many ways to highlight specifics or to focus the study on a single region. One example of this is weighting by patient number (rather than by study). As a result, large studies make a massive impact on the overall results, and the meta-analysis shifts to a more regional analysis of a single center as opposed to a global analysis of all modalities. Weighting by study enables the critical analysis of small population studies, including many from the West [27,37,43,50]. Due to the earlier adoption of ablative therapies, the East has a larger set of data in the early 1990s, whereas the West has a larger set in the 2000s.

A few weaknesses in the study are acknowledged. There are several limitations to the analysis, including missing data (OS, RFS, complication rate, local recurrence data, Child–Pugh score, age, number of patients who eventually underwent transplantation), study population, data inhomogeneity (related causes for HCC including underlying cirrhosis, median follow-up time), and inconsistent procedural methodology [30,34,50]. One particular metric listed above that would be valuable in analyzing efficacy trends would be liver function measurements, such as MELD/PELD score and Child–Pugh score. These are strong indicators of the underlying condition caused by HCC [68]. Additionally, many of the studies did not produce data about the equipment used, which increased the difficulty of making direct comparisons [18,23]. Large-scale RCTs must be performed in order to confirm the suggestions posited by this meta-analysis.

## 5. Conclusions

Interventional percutaneous ablative methods are useful tools that can be used in conjunction with surgical resection for the successful treatment of small-sized HCC. However, new techniques and protocols from surgeons are being developed and are greatly reducing the complication rates while increasing survival rates. These techniques are outside of the scope of this analysis comparing surgical resection to percutaneous ablation, but they should be noted, as these procedures will become a mainstay of surgical care for small-sized HCC. From 2006–2019, surgical resection produced the highest 5-year survival rates, which shows a drastic increase compared to the 1989–2005 timeframe. Microwave ablation and radiofrequency ablation are not significantly different in overall survival rates. Microwave ablation is the newest modality, and as a result, there are less data than the other two modalities studied in this meta-analysis. As MWA usage grows in prevalence, more quality data will be available to further study the comparative effectiveness of minimally invasive percutaneous and surgical treatments of small HCC.

## Figures and Tables

**Table 1:** Microwave Ablation Data.

Region	Number of Patients	Median Follow-Up Period (mo.)	Mean Tumor Size (cm)	Overall Survival Rates					Major Complication Rate (%)
				1-Year (%)	2-Year (%)	3-Year (%)	4-Year (%)	5-Year (%)	
Liang [4]	288	31.41		93	82	72	63	51	
Liang [5]	1007	38.0		91.2	72.5	59.8			
Jian-Ping [6]	293 113	29.3	2.6	98.1 98.0		73.7 82.0		48.2 46.9	3.2
Xu [7]	301	53.0	1.7	99.3		90.4		78.3	0.7
Thamtorawat [8]	129	11.8		91.3	81.7				2.2
Sun [9]	182		3.7	89	74	60			2.7
Ziemlewicz [10]	75	14.0 12.0	2.1 3.7	76					0
Baker [11]	219	10.9	3.2	80	61.5				3.2
Abdelaziz [12]	66		<5	91.6	86.1				3.2
Wang [13]	221	41.0	<5	87.1	63.2				3.8
Xu [14]	82		<5	92.7		63.4		41.1	3.7
Zhang [15]	77		<5	92.2		51.7		38.5	2.6
Yin [16]	220			95.5	89.1				
Total/Mean	3273	27.9		91.1	76.3	69.1	63.0	50.7	2.5

**Table 2:** Radiofrequency Data.

Region	Number of Patients	Median Follow-Up Period (mo.)	Mean Tumor Size (cm)	Overall Survival Rates					Major Complication Rate (%)
				1-Year (%)	2-Year (%)	3-Year (%)	4-Year (%)	5-Year (%)	
Yang [17]	382 88	28.0	3.4	84.3 92.5		54.4 60.3		41.2 43.2	4.9 0.8
Lee [18]	162	49.0	2.59	94.4		84.1		67.9	3.1
Osaki [19]	1161		<4	96.0		76.2		56.0	
Lencioni [20]	206	24.0	<5	97.0		71.0		48.0	
Tateishi [21]	319		<5	94.7	86.1	77.7	67.4	54.3	4.0
Chen [22]	256	53.0	1.7	83.3		66.9		41.2	2.4
Yoon [23]	570		<5	95.2	82.9	69.5	60.8	58.0	1.9
Livraghi [24]	218	31.0	<4					68.5	1.8
N’Kontc [25]	235	27.0	3.0			60.0		40.0	0.9
Shiina [26]	118	37.0	<4			81.0	74.0		3.2
Brunell [27]	70		<3			63.0			14
Huang [28]	115		<5	87.0	76.5	69.6	66.1	54.8	
Feng [29]	84		<4	93.1		83.1		67.2	
Viva [30]	79	15.6	<5	78		33			
Cho [31]	99		3.1	95.8		86.8		80	
Hiraok [32]	105		2.0			87.8		59.3	0
Yun [33]	255	42	2.1			92		87	
Hung [34]	190	14.5	2.4					67.4	
Nishi [35]	162	37	2.0	95.4		79.6		63.1	
Wang [36]	345		<3			80.3		72	
Peng [37]	71	59	1.2	90.5		70.9		62.1	19
Hasegawa [38]	5548	26	<3			81.0		61.1	
Total/Mean	10,838	32.3		91.2	81.8	72.8	67.1	59.6	3.4

**Table 3:** Surgical Resection Data.

Region	Number of Patients	Mean Tumor Size (cm)	Overall Survival Rates					Major Complication Rate (%)
			1-Year (%)	2-Year (%)	3-Year (%)	4-Year (%)	5-Year (%)	
Nagasue [39]	229	<4	80		51		26	24
Takenaka [40]	280	<5	88		70		50	50
Makuuchi [41]	352	<4	92		73		47	
Shimozawa [42]	135	<3	95		73		55	25
Franco [43]	72	<4	68		51			48
Belghiti [44]	300	<5	81		57		37	
Ercolani [45]	224	4.0	83		63		42	36.2
Cha [46]	164	<4	79		51		40	
Ringe [47]	131	<5			42.3		35.8	
Pitre [48]	153	<5			30.1		17.9	38.5
DiCarl [49]	122	<4			42.6		23.3	30
Lise [50]	100	<5					38	16
Grazi [51]	264	4.4			57		37	47
Zhou [1]	1000	<5					62.7	
Poon [52]	241	<5	82		62		49	
Kanem [53]	303		84		67		51	
Chen [22]	252		80		54.3		34.2	
Wayne [54]	249	<5	83				41.1	
Yeh [55]	218	<5	63		42		32	15.6
Capuss [56]	216				51		34	38.4
Natha [57]	788	3.2					39	
Yang [17]	260		87		56		38	
Yun [33]	215	2.1			98		94	
Hung [34]	229	2.9					79.3	
Wang [36]	260	<3			98		91.5	
Hasegawa [38]	5361	<3			85.3		71.1	
Peng [37]	74	1.1	98.5		87.7		71.9	51.4
Arii [58]	1318 502 2722 1548	<2 <2 2–5 2–5	96 92 95 95				72 56 58 45	
Total/Mean	18,282		85.3		61.9		49.0	35.0

**Table 4:** Combined Overall Survival and Complication Rates.

Modality	Number of Patients	Overall Survival Rates					Major Complication Rate (%)
		1-Year	2-Year	3-Year	4-Year	5-Year	
MWA	3725	91.1	76.3	69.1	63.0	50.7	3.2
RFA	10,838	91.2	81.8	72.8	67.1	59.6	2.6
SR	18,282	85.3		61.9		49.0	32.9

**Table 5:** Subgroup Analysis of Tumor Size.

MWA	1 Year Survival Rate (%)	3 Year Survival Rate (%)	5 Year Survival Rate (%)	Complication Rate (%)
<3 cm	92.9	82	57.8	2
<5 cm	91.1	69.1	50.7	2.5
3–5 cm	90.4	61.4	43.5	3.1
**RFA**	**1 year survival rate (%)**	**3 year survival rate (%)**	**5 year survival rate (%)**	**Complication rate (%)**
<3 cm	90.9	76.6	62.2	6.6
<5 cm	91.2	72.8	59.6	4.7
3–5 cm	91.4	68.6	55.6	2.5
**SR**	**1 year survival rate (%)**	**3 year survival rate (%)**	**5 year survival rate (%)**	**Complication rate (%)**
<3 cm	95.3	88.4	69.4	38.2
<5 cm	85.6	62.6	49.9	35
3–5 cm	80.3	54.0	39	34.4

## Data Availability

Not applicable.
